# Analysis of the urine flow characteristics inside catheters for intermittent catheter selection

**DOI:** 10.1038/s41598-024-64395-9

**Published:** 2024-06-10

**Authors:** Kyeongeun Lee, Jeongwon Han

**Affiliations:** College of Nursing Science, Kyung Hee University, Seoul, Republic of Korea

**Keywords:** Urology, Health care

## Abstract

In this study, we conducted a numerical analysis on catheter sizes using computational fluid dynamics to assess urinary flow rates during intermittent catheterization (IC). The results revealed that the fluid (urine) movement within a catheter is driven by intravesical pressure, with friction against the catheter walls being the main hindrance to fluid movement. Higher-viscosity fluids experienced increased friction with increasing intravesical pressure, resulting in reduced fluid velocity, whereas lower-viscosity fluids experienced reduced friction under similar pressure, leading to increased fluid velocity. Regarding urine characteristics, the results indicated that bacteriuria, with lower viscosity, exhibited higher flow rates, whereas glucosuria exhibited the lowest flow rates. Additionally, velocity gradients decreased with increasing catheter diameters, reducing friction and enhancing fluid speed, while the friction increased with decreasing diameters, reducing fluid velocity. These findings confirm that flow rates increased with larger catheter sizes. Furthermore, in terms of specific gravity, the results showed that a 12Fr catheter did not meet the ISO-suggested average flow rate (50 cc/min). The significance of this study lies in its application of fluid dynamics to nursing, examining urinary flow characteristics in catheterization. It is expected to aid nurses in selecting appropriate catheters for intermittent catheterization based on urinary test results.

## Introduction

Before performing intermittent catheterization (IC), nurses consider various factors such as the cause of voiding dysfunction, residual urine in the bladder, and the material, type, and structure of the catheter. The size of the catheter should be carefully selected because it can directly damage the urethra or cause problems with urine drainage^1^. Since IC is an invasive procedure, it may elevate the risk of trauma-induced infections if the catheter exerts continuous pressure on the bladder or urethra. Additionally, catheters themselves may cause inflammation through contact, emphasizing the need for appropriate sizing, especially in cases of residual urine or severe hematuria^2^. Repeated catheter insertions lead to urethral irritation and strictures; therefore, catheter with a right size should be selected^3,4^.

The American Urological Nurses Association recommends 6–12 Fr for children and 14–22 Fr for adults, while the European Association of Urology Nurses recommends 10–14 Fr for women and 12–14 Fr for men. However, studies suggest that catheter should be selected based on urine characteristics and density rather than solely based on physical conditions of patients^4,5^. A previous study conducted with nurses from the UK highlighted the variation in the required catheter size according to urine flow, recommending a 10 Fr catheter for initial female users voiding clear urine and a 16 Fr catheter for initial male users voiding slightly turbid or clear urine without clots^4^. Additionally, an 18 Fr catheter was recommended for cases with residual urine or blood clots, whereas 20–24 Fr was recommended for severe hematuria, post-prostatectomy, or bladder surgery. Another study recommended using a catheter that is 5 Fr smaller than the general adult size for patients with benign prostatic hyperplasia, while another study demonstrated that smaller catheter diameters obstructed urine flow^6,7^. A study conducted in Malaysia emphasized the importance of selecting optimal catheter size that rapidly expels urine while minimizing side effects, stressing the importance of urine properties and catheter dimensions for catheter selection^7^.

Over the years, individuals have increasingly practiced intermittent self-catheterization not only within medical facilities but also at home^8^. Previously, these individuals primarily sought education on cleanliness related to catheter use, but recently, the demand for knowledge on appropriate catheter length and size has surged^9^. This underscores the need for nurses to not only select suitable catheters for individuals but also provide education related to appropriate catheter selection. Improper catheter selection can increase the risks of infection, bleeding, trauma, posing significant safety concerns for patients^10^.

Conducting experiments that consider various variables, such as intravesical pressure (pressure from the bladder) and urine temperature, requires substantial economic and time resources and faces several limitations. Previous studies lacked substantial evidence and heavily relied on expert opinions or low-level research^9^, resulting in inconsistent recommendations among the national or professional bodies regarding catheter size^11^.

Fluid dynamics simulations of urine can enable the examination of urine flow rates and fluid characteristics through catheters of different sizes. Fluid dynamics is a scientific field focused on the application of the laws of motion to moving or stationary fluids^12^. This method enables the simulation of diverse scenarios and presents real phenomena more accurately by simultaneously incorporating multiple variables^13^. Additionally, it offers relative advantages in terms of result accuracy and experimental cost compared to traditional experimental designs^14^. Fluid dynamics has also been applied in medicine for visual representations essential for investigating vascular diseases or specific diagnoses^15^. There are inconsistencies in the recommended catheter size; for example, studies by the American Society of Mechanical Engineers suggested 14 Fr as the optimal catheter size using water instead of urine^16^, whereas Malaysian engineers recommended an 18 Fr catheter for efficient urine expulsion using the Navier–Stokes equation. However, this recommendation did not consider intravesical pressure and various urine atterns^17^.

Therefore, this study aims to provide fundamental data on IC for nurses. It seeks to explore urine flow rates based on different catheter sizes by considering various factors related to urination, such as intravesical pressure, urine temperature, and constituents. Additionally, it aims to examine the flow characteristics of urine within catheters. The goal of this study is to equip nurses with essential information to efficiently perform IC based on the findings related to urine discharge.

## Results

### Urinary flow rates based on the catheter size as a function of intravesical pressure, urine temperature, and constituent

**Figure 1:**
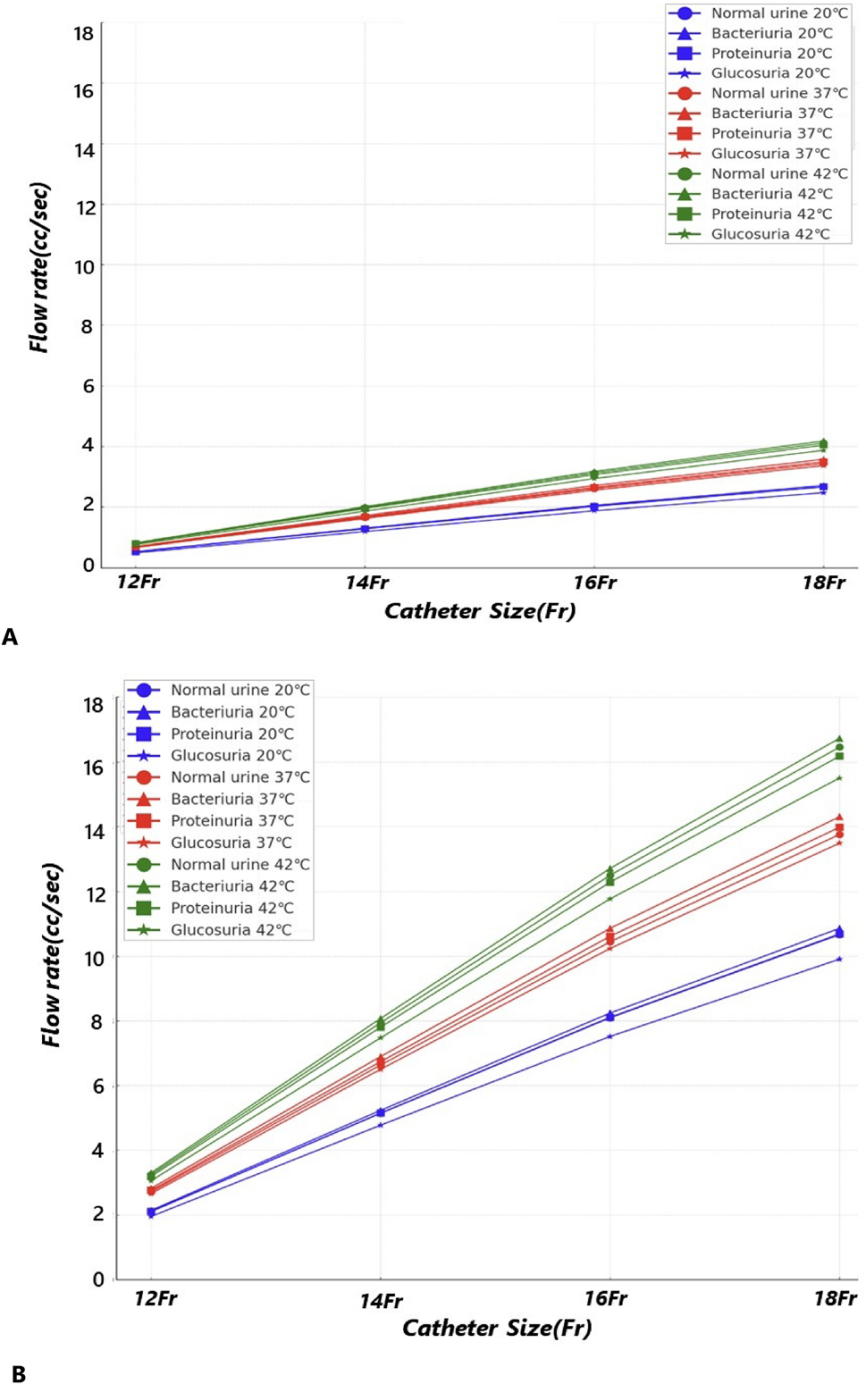
Urinary flow rates based on the catheter size as a function of intravesical pressure, urine temperature, and constituent. (**A**) Flow rates at different temperatures (20, 37, and 42 °C) for various catheter sizes (12, 14, 16, and 18 Fr) at minimum bladder pressure (5 cm H_2_O). (**B**) Flow rates at different temperatures (20, 37, and 42 °C) for various catheter sizes (12, 14, 16, and 18 Fr) at maximum bladder pressure (20 cm H_2_O).

Table 1 and Fig. 1 show the urine flow rates for different catheter sizes as a function of intravesical pressure, urine temperature, and constituent. In this study, the 12 and 18 Fr catheters exhibited the lowest and highest flow rates, respectively, when intravesical pressure, urine constituent, and urine temperature were kept constant. Additionally, with the same urine constituent, temperature, and catheter, higher intravesical pressures corresponded to higher urine flow rates. When the intravesical pressure, urine temperature, and catheter size were maintained, the presence of bacteria in the urine (bacteriuria) resulted in the highest flow rate, whereas glucose in the urine (glucosuria) exhibited the lowest flow rate. Higher urine temperatures were also associated with higher flow rates, given the intravesical pressure, urine constituent, and catheter size were constant. The specific range of urine flow rates by type is as follows. At a bladder pressure of 5 cm H_2_O, the flow rates were 0.526 (20 °C and 12 Fr) to 4.119 (42 °C and 18 Fr) cc/sec for normal urine, 0.535 (20 °C and 12 Fr) to 4.188 (42 °C and 18 Fr) cc/sec for bacteriuria, 0.527 (20 °C and 12 Fr) to 4.049 (42 °C and 18 Fr) cc/sec for proteinuria, and 0.488(20 °C and 12 Fr) to 3.879 (42 °C and 18 Fr) cc/sec for glucosuria. At a bladder pressure of 20 cm H_2_O, the flow rates were 2.103 (20 °C and 12 Fr) to 16.465 (42 °C and 18 Fr) cc/sec for normal urine, 2.141 (20 °C and 12 Fr) to 16.740 (42 °C and 18 Fr) cc/sec for bacteriuria, 2.107 (20 °C and 12 Fr) to 16.185 (42 °C and 18 Fr) cc/sec for proteinuria, and 1.954 (20 °C and 12 Fr) to 15.510 (42 °C and 18 Fr) cc/sec for glucosuria. The Reynolds number based on the results is as follows. At a bladder pressure of 5 cm H_2_O, Reynolds numbers were 313 (20 °C and 12 Fr) to 2,523 (42 °C and 18 Fr) for normal urine, 324 (20 °C and 12 Fr) to 2,609 (42 °C and 18 Fr) for bacteriuria, 314 (20 °C and 12 Fr) to 2,438 (42 °C and 18 Fr) for proteinuria, and 270 (20 °C and 12 Fr) to 2,237 (42 °C and 18 Fr) for glucosuria. At a bladder pressure of 20 cm H_2_O, Reynolds numbers were 1,251 (20 °C and 12 Fr) to 10,087 (42 °C and 18 Fr) for normal urine, 1,297 (20 °C and 12 Fr) to 10,428 (42 °C and 18 Fr) for bacteriuria, 1,256 (20 °C and 12 Fr) to 9,745 (42 °C and 18 Fr) for proteinuria, and 1,080 (20 °C and 12 Fr) to 8,945 (42 °C and 18 Fr) for glucosuria.

**Table 1:** Urinary flow rates based on the catheter size as a function of intravesical pressure, urine temperature, and constituent. (Flow rate: cc/sec).

Urine temperature	Urine constituent	5 cm H_2_O				20 cm H_2_O			
		12Fr	14Fr	16Fr	18Fr	12Fr	14Fr	16Fr	18Fr
20° C	Normal urine	0.526	1.285	2.024	2.667	2.103	5.142	8.096	10.670
	Bacteriuria	0.535	1.309	2.061	2.716	2.141	5.234	8.242	10.863
	Proteinuria	0.527	1.288	2.028	2.672	2.107	5.151	8.111	10.690
	Glucosuria	0.488	1.194	1.880	2.478	1.954	4.776	7.520	9.910
37° C	Normal urine	0.678	1.658	2.611	3.442	2.714	6.634	10.446	13.766
	Bacteriuria	0.705	1.724	2.715	3.578	2.822	6.897	10.861	14.312
	Proteinuria	0.689	1.685	2.654	3.497	2.758	6.741	10.615	13.988
	Glucosuria	0.665	1.626	2.560	3.374	2.660	6.504	10.241	13.496
42° C	Normal urine	0.812	1.985	3.126	4.119	3.248	7.941	12.502	16.465
	Bacteriuria	0.826	2.019	3.179	4.188	3.303	8.075	12.713	16.740
	Proteinuria	0.798	1.951	3.072	4.049	3.193	7.804	12.288	16.185
	Glucosuria	0.765	1.869	2.943	3.879	3.059	7.477	11.773	15.510

### Urinary flow rates based on the catheter size as a function of viscosity considering specific gravity

Table 2 shows the urine flow rates as a function of viscosity, considering specific gravity. The urine viscosity increased with an increase in specific gravity. Additionally, urine flow rates increased with higher urine temperatures, higher intravesical pressures, and larger catheter sizes. The specific range of urine flow rate is as follows. At a bladder pressure of 5 cmH_2_O, the flow rates were 0.449 to 2.919 cc/sec at 20 °C, 0.596 to 3.770 cc/sec at 37 °C, and 0.732 to 4.448 cc/sec at 42 °C. At a bladder pressure of 20 cm H_2_O, the flow rates were 1.798 to 11.674 cc/sec at 20 °C, 2.382 to 15.060 cc/sec at 37 °C, and 2.928 to 17.736 cc/sec at 42 °C. The Reynolds number based on the results is as follows. At a bladder pressure of 5 cmH2O, Rynolds numbers were 228 to1,267 at 20 °C, 402 to 2,113 at 37 °C, and 606 to 2,943 at 42 °C. At a bladder pressure of 20 cmH2O, Rynolds numbers were 914 to 5,067 at 20 °C, 1,605 to 8,442 at 37 °C, and 2,426 to 11,736 at 42 °C.

**Table 2:** Urinary flow rates based on the catheter size as a function of the viscosity considering the specific gravity. (Flow rate: cc/sec).

Urine temper-ature	Urine specific gravity	Visco-sity (cSt)	5 cm H_2_O				20 cm H_2_O			
			12Fr	14Fr	16Fr	18Fr	12Fr	14Fr	16Fr	18Fr
20° C	1.016	0.9779	0.575	1.407	2.215	2.919	2.301	5.626	8.859	11.674
	1.017	1.0464	0.538	1.314	2.070	2.728	2.151	5.258	8.279	10.911
	1.018	1.0807	0.521	1.274	2.006	2.644	2.085	5.096	8.024	10.576
	1.019	1.1150	0.505	1.234	1.942	2.560	2.019	4.934	7.770	10.240
	1.020	1.1835	0.475	1.162	1.830	2.412	1.902	4.649	7.320	9.647
	1.021	1.2178	0.462	1.130	1.780	2.346	1.850	4.521	7.119	9.383
	1.022	1.2520	0.449	1.099	1.730	2.280	1.798	4.394	6.919	9.119
37° C	1.016	0.7571	0.743	1.817	2.861	3.770	2.973	7.266	11.438	15.060
	1.017	0.8040	0.700	1.711	2.694	3.550	2.799	6.842	10.774	14.197
	1.018	0.8275	0.680	1.664	2.619	3.452	2.722	6.654	10.478	13.810
	1.019	0.8509	0.661	1.616	2.545	3.355	2.645	6.466	10.182	13.422
	1.020	0.8978	0.627	1.532	2.412	3.179	2.507	6.128	9.649	12.721
	1.021	0.9213	0.611	1.494	2.352	3.100	2.444	5.975	9.409	12.405
	1.022	0.9447	0.596	1.456	2.292	3.021	2.382	5.823	9.170	12.088
42° C	1.016	0.6414	0.877	2.144	3.376	4.448	3.508	8.575	13.485	17.736
	1.017	0.6732	0.836	2.043	3.217	4.240	3.343	8.172	12.866	16.950
	1.018	0.6891	0.817	1.997	3.145	4.145	3.268	7.988	12.580	16.583
	1.019	0.7050	0.798	1.951	3.072	4.049	3.193	7.804	12.294	16.216
	1.020	0.7367	0.764	1.867	2.940	3.875	3.055	7.467	11.766	15.531
	1.021	0.7526	0.748	1.828	2.879	3.794	2.992	7.313	11.523	15.213
	1.022	0.7685	0.732	1.790	2.818	3.714	2.928	7.159	11.279	14.895

### Characteristics of the urine flow in intermittent catheter

**Figure 2:**
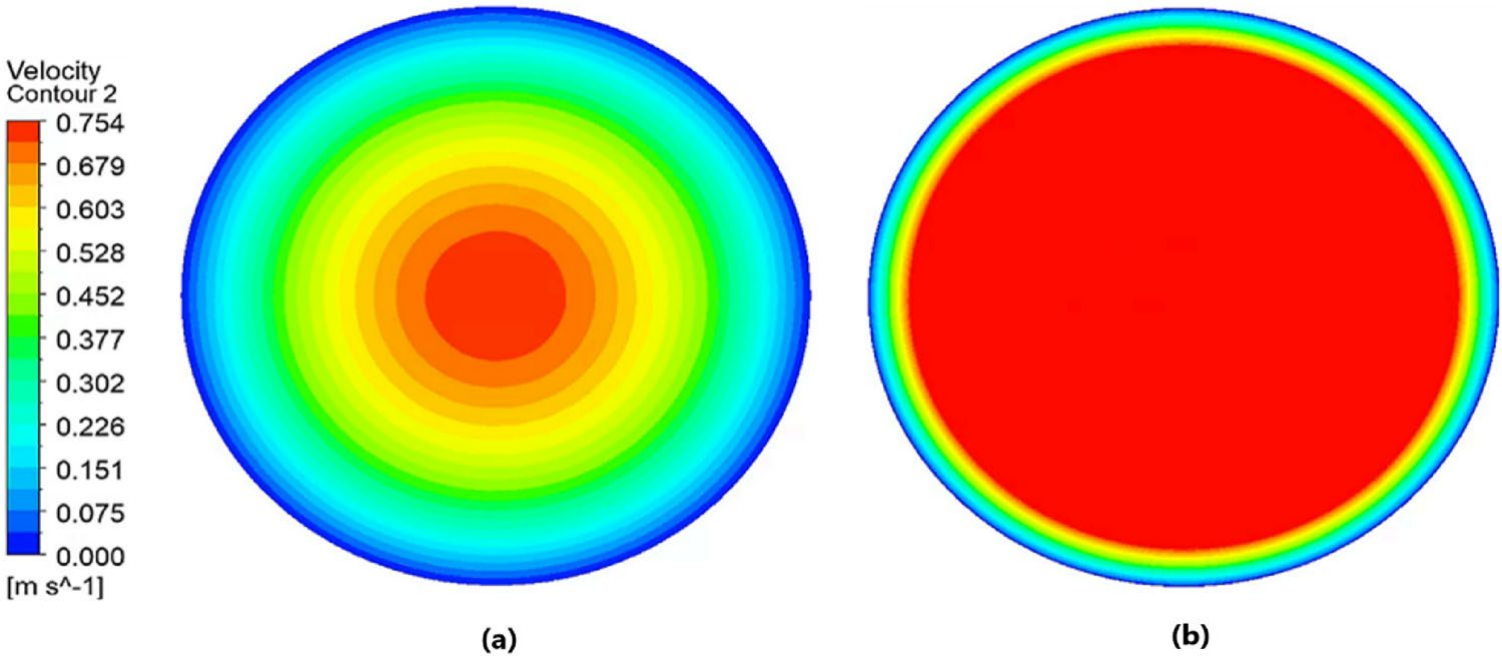
Velocity contour lines on the cross-section of the catheter due to intravesical pressure in normal urine passing through an 18 Fr catheter at 20 °C. (**a**) 5 cm H_2_O and (**b**) 20 cm H_2_O.

The fluid dynamics of urine, based on the analysis of the intravesical pressure, urine temperature, urine characteristics, and urethral catheter size using computational fluid dynamics (CFD), are summarized here. The movement of urine within the urethral catheter is primarily induced by the pressure generated within the bladder. As bladder pressure increases, the flow rate and velocity of urine through the catheter also increase(Fig. 2). When laminar flow is fully developed, the flow rate and bladder pressure are theoretically directly proportional. Although the flow observed in this study is not an ideal fully developed laminar flow, the relationship between flow rate and intravesical pressure can be considered nearly linear, given that the driving force of the flow is the intravesical pressure.

**Figure 3:**
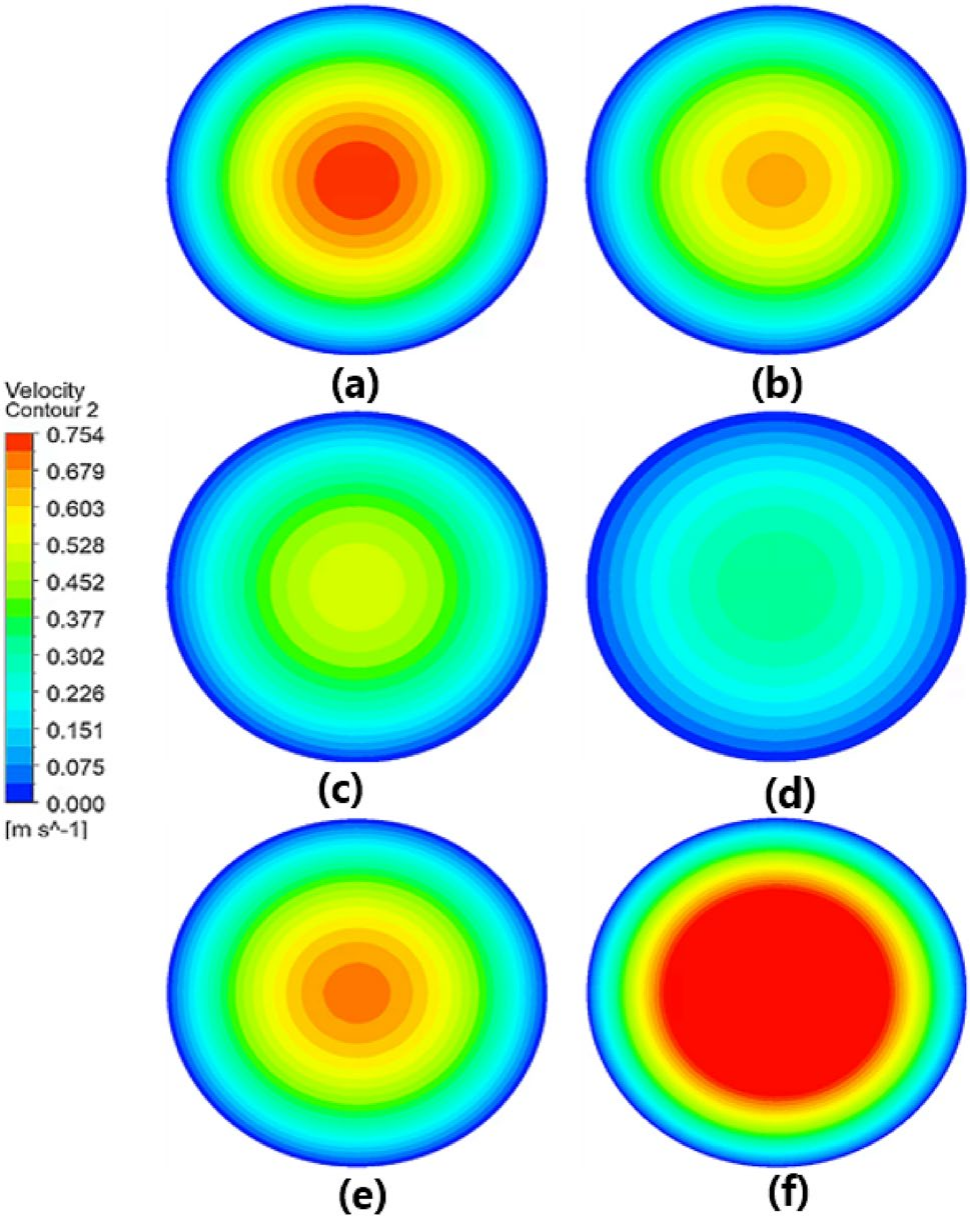
Velocity contour lines on the cross-section view of the catheter at the midpoint of its length at an intravesical pressure of 5 cm H_2_O. Normal urine passing through an (**a**) 18Fr, (**b**) 16Fr, (**c**) 14Fr, and (**d**) 12Fr catheters at 20 °C. (**e**) Glucose urine passing through an 18Fr catheter at 20 °C. (**f**) Normal urine passing through an 18Fr catheter at 42 °C.

The disruption of fluid movement can be attributed to the frictional force exerted on the catheter walls. The magnitude of this frictional force ($\tau$) is represented as follows:

1 $$ \tau = \left| {\mu \frac{dv}{{dr}}} \right|_{r = R} , $$

where $\mu$ is the dynamic viscosity, $v$ is the velocity component perpendicular to the catheter section, $r$ is the radial component in polar coordinates, and $R$ is the radius of the catheter. As illustrated in Eq. 1, the magnitude of the frictional force increases linearly with an increase in $\mu$ and the intensity of the velocity gradient at the catheter wall (Fig. 3a, e, f). This implies that for fluids with higher viscosity, under the same intravesical pressure, frictional force increases, leading to reduced fluid velocity. Conversely, for fluids with lower viscosity, the frictional force decreases, resulting in increased fluid velocity. Moreover, an increase in the catheter diameter reduces the velocity gradient at the catheter wall, thereby decreasing the frictional force and increasing the fluid velocity. In contrast, a decrease in the diameter leads to an increase in the frictional force and a decrease in fluid velocity (Fig. 3a–d).

## Discussion

In this study, we utilized CFD simulations based on the Navier–Stokes equations to analyze urine flow in intermittent catheters under varying intravesical pressure, urine temperature, and urinary constituents, considering different catheter sizes. The key findings are discussed below.

First, when urine constituents, urine temperature, and catheter size were maintained, urine flow within the catheter increased with increasing intravesical pressure. This observation aligns with those reported in previous research indicating a correlation between increased intravesical pressure and accelerated urine flow during voiding^18^. As the bladder volume increases, intravesical pressure surpasses resistance to urine flow, triggering the voiding reflex, underscoring the importance of intravesical pressure in urine discharge^19,20^. In clinical practice, nurses educate individuals with voiding difficulties, recommending periodic IC or self-catheterization to maintain bladder volume below 400 ml at intervals of 3–6 h, facilitating bladder emptying and resolving voiding issues^21^. However, individuals with neurogenic bladder disorders may experience inadequate voiding due to low intravesical pressure during self-catheterization, resulting in residual urine and increased risks of complications such as cystitis, pyelonephritis, or sepsis^22^. Additionally, alterations in bladder pressure can occur due to lower urinary tract strictures in adult males or posterior urethral valve (PUV) disorders in children. Particularly in children with PUV disorders, significant changes in urinary flow can be observed after endoscopic valve ablation compared to that in pre-surgery conditions^23–25^. Therefore, nurses should be educated about the changes in urine flow related to these conditions. Moreover, nurses should educate individuals who require self-catheterization due to various diseases, emphasizing catheter selection, management, and symptom monitoring, with a recommendation to visit a healthcare facility if necessary. Nurses should also explain voiding mechanisms so that individuals can perform self-catheterization accurately and safely. They should advise patients to consider potential bladder pressure issue or urinary tract problems associated with their conditions if urine does not flow normally^26,27^ This study provides pertinent information to both nurses and patients in this aspect. In clinical practice, nurses typically perform IC with individuals lying down, while individuals undergoing aseptic IC typically perform it while sitting^28,29^. When the bladder and urethra transition from a horizontal to a vertical position, the force supporting the abdominal organs significantly increases the intravesical pressure, with the pressure often being 2–3 times higher than that in the horizontal position^28,30^. Nurses can educate individuals performing self-catheterization about the effects of different postures on the voiding time. They should explain that adopting a sitting or standing posture can reduce voiding time due to the pressure exerted during urination.

Second, the results indicate that when the intravesical pressure, urinary constituents, and urethral size are maintained, urine flow increases with an increase in urine temperature. This finding aligns with those from studies that examined the variability in urinary viscosity and reported a decrease in viscosity with increasing urine temperature. This phenomenon is related to the micturition process and is consistent with conclusions drawn in previous literature^29^. Specifically, the urine flow at 42 °C was over 1.5 times higher than that at 20 °C. While previous research primarily analyzed urine at room temperature, making precise comparisons challenging, this study confirms that the fluid viscosity changes with temperature, thereby altering flow and velocity of the fluid^31^. In clinical settings, therapeutic interventions such as hypothermia or hyperthermia can be applied based on the health status and conditions. These interventions can affect the temperature of the final excreted product, i.e., urine, due to temperature changes in the kidneys and bladder^32,33^. However, individuals undergoing hypothermia treatment often tend to maintain an indwelling catheter rather than performing IC. Therefore, based on previous studies, this study establishes a limitation by setting the standard urine temperature for hypothermic states at 20 °C. Our findings suggest that individuals requiring ongoing IC may experience relatively increased voiding times in environments where their body temperature decreases. Consequently, corresponding voiding information could be provided to these individuals. Conversely, nurses can consider using smaller catheter sizes than usual for those individuals who have high fever and are undergoing IC, considering the possibility of increased urinary flow due to repeated catheterization, which may lead to urethral mucosal damage.

Third, when intravesical pressure, urine temperature, and urethral size were maintained, bacteriuria exhibited the highest urine flow and the lowest glucose content. Although previous studies exploring urine flow as a function of its constituents are limited, making precise comparisons challenging, the higher urine flow in bacteriuria compared to that in normal urine flow aligns with the results obtained in previous research. This research confirms a proportional relationship between glucose concentration and flow rate by adding glucose solution to urine^34^. Additionally, studies investigating the relationship between urine flow rate and blood flow velocity have shown that in patients with high blood sugar, urine viscosity increases due to sugar, which is consistent with our findings^35^. Bacteriuria is characterized by increased turbidity or cloudiness compared to normal urine, often due to white blood cells or bacteria^36^. Previous studies suggested that bacteria interact with other cells while moving within a liquid, enhancing the flow of nutrients or promoting the collective movement of suspended particles, which might induce an increase in the flow rate^37^. The increased flow rate could be attributed to the exertion of force by bacteria through flagellar movement in the surrounding fluid, favorable viscosity in high-resistance environments, or the formation of turbulent flows as bacteria aggregate, leading to faster movement and increased flow rate. These findings are consistent with previous research^38^. In clinical practice, nurses should consider using larger catheter sizes than the usual ones for individuals with hyperglycemia due to the anticipated decrease in urine flow. For patients with bacteriuria experiencing discomfort or undergoing repeated catheterization, smaller catheter sizes might alleviate discomfort. However, since this study applied constituent analysis to mild states, exploring urine information collected from repeated studies and diverse individuals is necessary to validate these findings.

Fourth, our findings indicated an increase in the urine flow with an increase in catheter size. This aligns with previous research on urine flow as a function of catheter size, suggesting that larger catheters lead to increased urine flow^39^. This simulation analysis revealed that an increase in catheter diameter reduces the velocity gradients on the catheter wall, thus reducing friction and resulting in an increased flow rate. Conversely, a decrease in diameter increases friction, leading to decreased flow rate. Nurses might consider selecting smaller catheters to minimize urethral damage at the onset of catheterization. However, longer catheter retention might increase the risk of the catheter pressing against the urethra wall, obstructing sweat gland drainage, and causing inflammation, including urinary tract infections such as urethritis^40^. Insertion complications, such as twisting or the formation of strictures, may increase the risk of urethral damage^40^. Excessively large catheters may cause discomfort and stimulate the urethral mucosa, leading to ischemia and urethral stricture^4^. Moreover, damage to the bladder urothelium and urethral walls can increase the risk of infection. Discomfort and pain during catheter insertion might deter regular catheterization, reducing the quality of life for individuals performing clean IC^10^. Proper catheter selection is crucial, as discomfort and pain during catheter insertion or positioning are known to significantly decrease the quality of life for self-clean IC patients^9^. In clinical practice, nurses often perform IC to obtain 30–50 ml of sterile urine for various tests, including urine culture. Although guidelines recommend minimizing catheterization due to increased infection and damage risks, in cases where sterile sample collection is not feasible, nurses must perform the procedure for accurate testing^41^. Selecting a smaller catheter size to collect a small amount of urine for testing purposes could mitigate the risk of damage while obtaining the required sample volume for accurate testing.

Fifth, our findings revealed that the urine viscosity increased as specific gravity increased. When analyzing flow while considering both specific gravity and viscosity, a 12 Fr catheter did not meet the average flow rate (50 cc/min) specified by ISO standards. In clinical practice, urine specific gravity is often used to assess a patient’s condition rather than urine viscosity^42^. Therefore, for clinical applications of urinary flow rate information, data based on viscosity associated with specific gravity is crucial. Although most previous studies analyzing urine flow primarily focused on viscosity, it is challenging to precisely compare their findings with the results of this study. Specific gravity directly reflects the urine concentration^39,43^, which is linearly related to increased viscosity and can be useful in predicting urine flow^44^. Specific gravity serves as a convenient measure to evaluate hydration status, kidney function, and conditions such as urinary incontinence, providing essential insights for intermittent catheter selection and predicting urine flow rates^45,46^. However, most prior research approached flow rate analysis by solely considering viscosity, thereby limiting practical clinical application. This study also has limitations, such as the limited presentation of urine viscosity range based on specific gravity and focusing solely on viscosity changes associated with increased specific gravity in normal urine. Nonetheless, the significance of this study lies in highlighting the need for flow analysis considering the easily accessible specific gravity for nurses to more accurately utilize IC and conduct self-catheterization education. Additionally, although Table 2 illustrates how viscosity changes despite identical specific gravity due to body temperature, further research on temperature-specific gravity and comprehensive viscosity presentation could assist nurses in selecting appropriate intermittent catheters in clinical settings.

Individuals undergoing IC should receive prescriptions for various catheter sizes based on their unique characteristics and situations, enabling them to select the most suitable and safe self-catheterization method. Nurse education on this diversity in catheter selection would facilitate smoother self-catheterization practices, including IC, within households.

## Conclusion

We conducted CFD simulations using the Navier–Stokes equations to analyze urinary flow rates in an intermittent catheter, considering intravesical pressure, urine temperature, and urine constituents. This research aims to provide fundamental data for selecting the appropriate catheter sizes. The results indicated that higher intravesical pressure led to increased urinary flow within the catheter, when urine constituents, temperature, and catheter size were maintained. Furthermore, higher urine temperatures correlated with greater flow rates when intravesical pressure, urine constituents, and catheter size were maintained. Bacteriuria exhibited the highest flow rate, whereas glucosuria exhibited the lowest flow rate under constant intravesical pressure, urine temperature, and catheter size. Additionally, the flow rate increased as the intermittent catheter size increased. An increase in specific gravity was associated with increased urine viscosity, which is consistent with previous findings. When both specific gravity and viscosity were considered simultaneously, a 12 Fr catheter did not achieve the average flow rate (50 cc/min) specified by ISO standards. This study is significant as it integrates fluid mechanics into nursing research, exploring ways for nurses to perform IC effectively, while reducing patient discomfort. Traditionally, such research has been possible only with real patients in nursing studies. However, this study utilizes CFD to accurately simulate various clinical scenarios. A limitation of this study is the assumption that the IC is a rigid body rather than a deformable one; in addition, the impact of catheter dimensions on the urethra walls was neglected. Subsequent research should aim to establish diverse data collection methods considering urine constituents and individual subjects, thereby directly proposing catheter sizes based on these considerations.

## Methods

### Study design

A numerical analysis was conducted to investigate the flow characteristics within the catheter during intermittent urination and to analyze the urinary flow rate. There was no human or animal involvement in this study.

### Parametric study

We analyzed the flow characteristics through a catheter based on various urine properties that vary according to the health problems of the patients, bladder pressure, and urine temperature, as well as changes in the diameter of the catheter. With the length of the catheter fixed, the analysis was implemented based on the diameter of the catheter. Analyses were carried out while varying the conditions (urine properties, bladder pressure, and urine temperature) on the implemented geometry.

### Subject of interpretation

**Figure 4:**
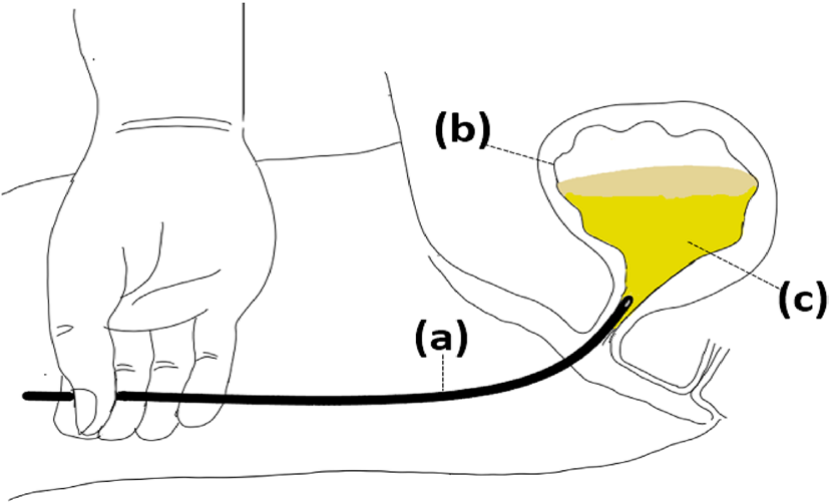
IC system. (**a**) Catheter size: defined as the inner diameter sizes of 12–18 Fr catheters. (**b**) Intravesical pressures observed in adults in a supine position at rest: minimum pressure is 5 cm H_2_O (490.319 Pa) and maximum pressure is 20 cm H_2_O (1961.276 Pa). (**c**) Urine classification: categorized by viscosity and specific gravity of urine type (normal urine, bacteriuria, proteinuria, and glucosuria). Urine temperature: 20, 37, and 42 °C. Length of catheters: set at 340 mm.

The subject comprises of a system structure consisting of intravesical pressure, urine temperature, urine constituent, and intermittent catheter size, as shown in Fig. 4.

### Intravesical pressure

Intravesical pressure was applied based on the minimum (5 cm H_2_O) and maximum (20 cm H_2_O) pressures observed in adults in a supine position at rest^47^.

### Urine temperature

Fluid viscosity and specific gravity vary with temperature; hence, based on previous research, urine temperature was set at three benchmarks: low body temperature of 20 °C, normal human body temperature of 37 °C, and feverish state temperature assumed at 42° C^28^.

### Urine classification

Urine patterns were categorized into normal, bacteriuria, proteinuria, and glucosuria based on preceding research that provided the viscosity and specific gravity results of urine, which are essential for interpreting the study^28^.

### Intermittent catheter size (internal diameter)

The intermittent catheter sizes selected for intermittent catheterization in adults primarily include 12 Fr (2.0 mm), 14 Fr (2.5 mm), 16 Fr (2.8 mm), and 18 Fr (3.0 mm).

### Data collection

Data were collected through a literature review and information provided by the manufacturer. The information regarding the catheter was obtained from an ISO-compliant company that manufactures catheters (ISO 213485:2016). Medical devices in Korea are used in clinical settings only if they meet ISO standards. Therefore, the provided information is based on the ISO standards rather than specific company details. The specific parameters are described below.

### Intravesical pressure

It is defined as the pressure within the bladder in adults during a stable, supine position. The minimum pressure was set at 490.319 Pa (5 cm H_2_O) and the maximum pressure was set at 1961.276 Pa (20 cm H_2_O)^47^.

### Viscosity and specific gravity based on urine constituent

Viscosities were determined based on preceding research, considering urine constituent. For normal urine, viscosities were defined as: at 20 °C, 1.0700 centistokes (cSt); 37 °C, 0.8293 cSt; and 42 °C, 0.6928 cSt. For bacteriuria, viscosities were defined as: 20 °C, 1.0512 cSt; 37 °C, 0.7976 cSt; and 42 °C, 0.6813 cSt. For proteinuria, viscosities were defined as: 20 °C, 1.0677 cSt; 37 °C, 0.8161 cSt; and 42 °C, 0.7049 cSt. For glucosuria, viscosities were defined as: 20 °C, 1.1520 cSt; 37 °C, 0.8459 cSt; and 42 °C, 0.7359 cSt. Furthermore, considering the correlation between urine specific gravity and viscosity from previous studies, the study analyzed viscosity based on the specific gravity according to the temperature ranges: for specific gravity range of 1.016–1.022 at 20 °C, viscosity ranged between 0.9779 and 1.2520 cSt; for specific gravity range of 1.016–1.022 at 37 °C, viscosity ranged between 0.7571 and 0.9447 cSt; and for specific gravity range of 1.016–1.022 at 42 °C, viscosity ranged between 0.6414 and 0.7685 cSt^28^.

### Internal diameter and length of intermittent catheters

Intermittent catheter lengths were determined based on the ISO information (ISO 213485:2016). For the inner diameter of the catheters, considering restrictions on external information disclosure by manufacturers and in alignment with the research topic and objectives, we utilized information obtained from one company in South Korea (Sewoon Medical Co., Ltd). The data were acquired after explaining the research purpose and obtaining permission for data utilization. The inner diameters were set as follows for each size: 12 Fr (2.0 mm), 14 Fr (2.5 mm), 16 Fr (2.8 mm), and 18 Fr (3.0 mm). The lengths of all catheters were set to 340 mm.

### Data analysis

#### Modeling of intermittent catheters

The urethral catheter, representing the main urine flow path, was modeled using Ansys SpaceClaim software (Ansys, Inc, USA). The length of the urethral catheter was uniformly set to 340 mm, and the inner diameter of the catheter was modeled. The shape of the object analyzed is a circular cylinder.

#### Grid configuration

**Figure 5:**
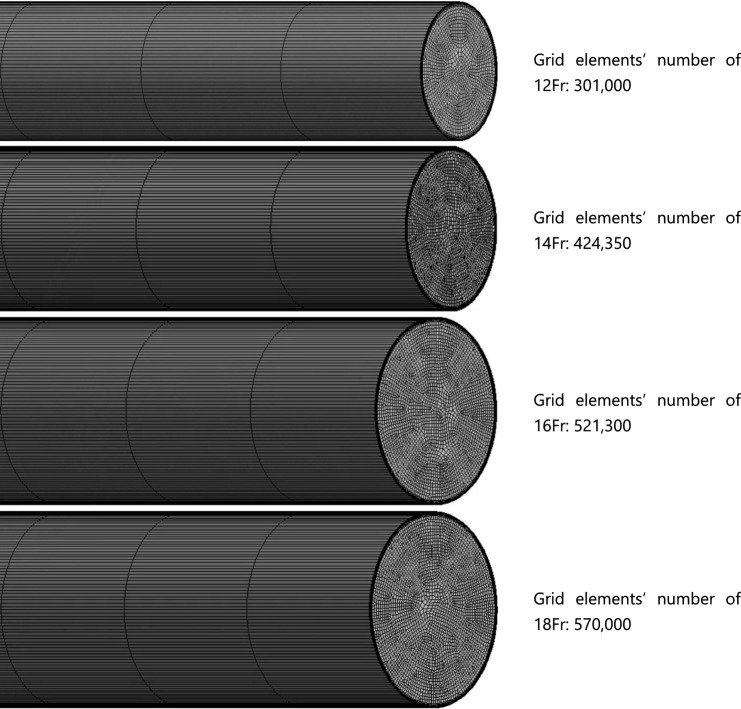
Grid systems used for CFD analysis.

After modeling the urethral catheter, the flow analysis domain was divided into a collection of small volumes (grid system) to apply the Navier–Stokes equations. The Ansys Meshing program (Ansys, Inc, USA) was employed for grid generation. To ensure accuracy, the minimum grid size at the catheter wall, where the velocity gradient is the highest, was set at 5.0 × 10^−6^ m, creating a dense grid system with seven layers on each of the seven walls. Inside the urethral catheter, a grid structure of square or triangular prisms was created to enhance the accuracy and convergence. The total number of grid elements used for the analysis ranged from approximately 301,00 to 570,000 (Fig. 5).

#### Configuration of parameters for simulation

**Figure 6:**
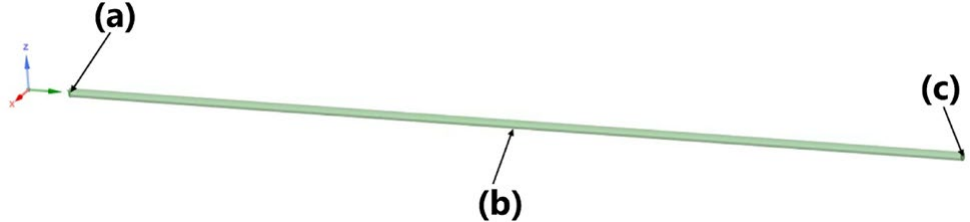
Boundary conditions used for the analysis. (**a**) Outlet boundary condition, atmosphere pressure. (**b**) No-slip boundary condition. (**c**) Inlet boundary condition, intravesical pressure (5 cm H_2_O or 20 cm H_2_O).

The Ansys CFX program (Ansys, Inc, USA) was utilized to compute the three-dimensional (3D), incompressible, steady-state Navier–Stokes equations along with the continuity equation. Urine was assumed to be a Newtonian fluid. The density and viscosity of the working fluid were set according to the urine type. Dirichlet boundary conditions were set at the inlet, while opening boundary conditions were set at the outlet to allow urine to exit without resistance. The catheter wall was set with a no-slip condition. The pressure at the inlet was set to intravesical pressure, while that at the outlet was set to atmospheric pressure (Fig. 6).

#### Running the simulation

Simulation was performed using computer hardware components, such as memory, central processing unit, and graphics processing unit, to conduct calculations. The completed analysis utilized the result files to extract and analyze necessary data, including pressure distribution within the urethral catheter, velocity distribution of urine, and urine flow rate within the catheter.

## Data Availability

The datasets used and/or analyzed during the current study available from the corresponding author on reasonable request.
